# Redetermination of hexa­sodium hepta­molybdate(VI) 14-hydrate

**DOI:** 10.1107/S1600536810012316

**Published:** 2010-04-10

**Authors:** Lujiang Hao, Jiangkui Chen, Xiaofei Zhang

**Affiliations:** a College of Food and Biological Engineering, Shandong Institute of Light Industry, Jinan 250353, People’s Republic of China

## Abstract

The structure of the title compound, Na_6_(Mo_7_O_24_)·14H_2_O, has been redetermined [Sjöbom & Hedman (1973). *Acta Chem. Scand.* **27**, 3673–3674] and the hydrogen atoms have been located. The Na^+^ cations adopt distorted octa­hedral geometries and the structure of the [Mo_7_O_24_]^6−^ anion is consistent with those of other hepta­molbydates. In the crystal, numerous O—H⋯O hydrogen bonds help to establish the packing.

## Related literature

For general background to polyoxometalates, see: Pope & Müller (1991 ▶). For polyoxometalates reported by our group, see: Zhang, Dou *et al.* (2009 ▶); Zhang, Wei *et al.* (2009 ▶). For the structures of other [Mo_7_O_24_]^6−^ heteropolyanions, see: Evans *et al.* (1975 ▶); Yang *et al.* (2002 ▶). For the previous determination of the title compound, see: Sjöbom & Hedman (1973 ▶). For Na—O bond lengths, see: Turpeinen *et al.* (2001 ▶); An *et al.* (2004 ▶).
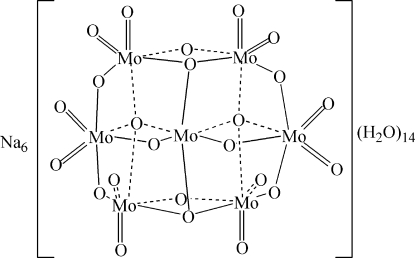


## Experimental

### 

#### Crystal data

Na_6_(Mo_7_O_24_)·14H_2_O
*M* *_r_* = 1445.74
Orthorhombic, 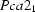

*a* = 21.1304 (2) Å
*b* = 10.3733 (1) Å
*c* = 15.6094 (2) Å
*V* = 3421.46 (6) Å^3^
*Z* = 4
Mo *K*α radiation
μ = 2.68 mm^−1^
*T* = 296 K
0.12 × 0.10 × 0.08 mm

#### Data collection

Bruker APEXII CCD diffractometer
Absorption correction: multi-scan (*SADABS*; Bruker, 2001 ▶) *T*_min_ = 0.739, *T*_max_ = 0.814
16564 measured reflections
5831 independent reflections
5748 reflections with *I* > 2σ(*I*)
*R*_int_ = 0.021

#### Refinement

*R*[*F* ^2^ > 2σ(*F* ^2^)] = 0.017
*wR*(*F* ^2^) = 0.044
*S* = 1.00
5831 reflections
545 parameters
63 restraints
H atoms treated by a mixture of independent and constrained refinement
Δρ_max_ = 1.12 e Å^−3^
Δρ_min_ = −1.00 e Å^−3^
Absolute structure: Flack (1983 ▶), 2689 Friedel pairs
Flack parameter: −0.02 (2)

### 

Data collection: *APEX2* (Bruker, 2004 ▶); cell refinement: *SAINT-Plus* (Bruker, 2001 ▶); data reduction: *SAINT-Plus*; program(s) used to solve structure: *SHELXS97* (Sheldrick, 2008 ▶); program(s) used to refine structure: *SHELXL97* (Sheldrick, 2008 ▶); molecular graphics: *SHELXTL* (Sheldrick, 2008 ▶); software used to prepare material for publication: *SHELXTL*.

## Supplementary Material

Crystal structure: contains datablocks global, I. DOI: 10.1107/S1600536810012316/hb5372sup1.cif

Structure factors: contains datablocks I. DOI: 10.1107/S1600536810012316/hb5372Isup2.hkl

Additional supplementary materials:  crystallographic information; 3D view; checkCIF report

## Figures and Tables

**Table 1:** Hydrogen-bond geometry (Å, °)

*D*—H⋯*A*	*D*—H	H⋯*A*	*D*⋯*A*	*D*—H⋯*A*
O6*W*—H12*W*⋯O3	0.83 (4)	2.04 (2)	2.804 (5)	156 (5)
O9*W*—H17*W*⋯O6	0.82 (1)	2.37 (1)	3.182 (6)	168 (6)
O12*W*—H23*W*⋯O8	0.82 (5)	2.10 (5)	2.920 (6)	176 (5)
O12*W*—H24*W*⋯O6	0.82 (4)	2.73 (7)	3.069 (6)	106 (6)
O1*W*—H1*W*⋯O11*W*^i^	0.82 (1)	1.93 (1)	2.747 (7)	175 (7)
O2*W*—H3*W*⋯O14*W*^ii^	0.82 (3)	2.06 (1)	2.865 (7)	168 (5)
O2*W*—H4*W*⋯O24^iii^	0.82 (2)	2.37 (7)	3.018 (6)	136 (8)
O3*W*—H5*W*⋯O8*W*^iii^	0.83 (4)	2.16 (6)	2.760 (6)	129 (6)
O4*W*—H7*W*⋯O14*W*^ii^	0.82 (3)	2.04 (3)	2.850 (6)	169 (5)
O4*W*—H8*W*⋯O7*W*^iii^	0.82 (3)	2.06 (2)	2.872 (6)	168 (9)
O5*W*—H9*W*⋯O23^iv^	0.82 (2)	2.41 (6)	2.937 (6)	123 (6)
O5*W*—H10*W*⋯O12^v^	0.83 (6)	2.45 (4)	3.179 (6)	149 (8)
O6*W*—H11*W*⋯O14^v^	0.82 (3)	2.15 (3)	2.874 (5)	147 (4)
O7*W*—H13*W*⋯O12^v^	0.82 (3)	2.11 (3)	2.878 (5)	157 (6)
O7*W*—H13*W*⋯O11^v^	0.82 (3)	2.55 (5)	3.036 (5)	119 (5)
O7*W*—H14*W*⋯O5^vi^	0.82 (6)	2.00 (5)	2.812 (6)	168 (9)
O8*W*—H15*W*⋯O14*W*^i^	0.82 (2)	2.13 (3)	2.868 (6)	149 (5)
O8*W*—H16*W*⋯O4^vi^	0.83 (5)	2.05 (4)	2.808 (5)	154 (10)
O9*W*—H18*W*⋯O15^v^	0.82 (3)	2.21 (3)	3.021 (6)	172 (6)
O10*W*—H19*W*⋯O13*W*^i^	0.82 (3)	2.06 (3)	2.877 (7)	176 (6)
O10*W*—H20*W*⋯O7^vi^	0.82 (2)	2.10 (2)	2.862 (5)	154 (5)
O11*W*—H21*W*⋯O1^vii^	0.82 (2)	1.98 (3)	2.781 (5)	167 (5)
O11*W*—H22*W*⋯O23^v^	0.83 (4)	2.03 (3)	2.771 (5)	150 (6)
O13*W*—H25*W*⋯O20^iv^	0.82 (3)	2.55 (5)	2.918 (6)	109 (4)
O13*W*—H26*W*⋯O14^v^	0.81 (5)	2.23 (3)	2.935 (6)	144 (5)
O14*W*—H27*W*⋯O21^viii^	0.82 (1)	1.87 (2)	2.662 (5)	163 (6)
O14*W*—H28*W*⋯O8^ix^	0.82 (4)	1.96 (2)	2.761 (5)	167 (9)

Symmetry codes: (i) 
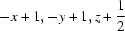
; (ii) 
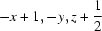
; (iii) 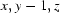; (iv) 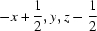; (v) 
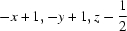
; (vi) 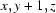; (vii) 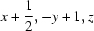; (viii) 
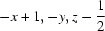
; (ix) 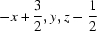.

**Fig. 1.**
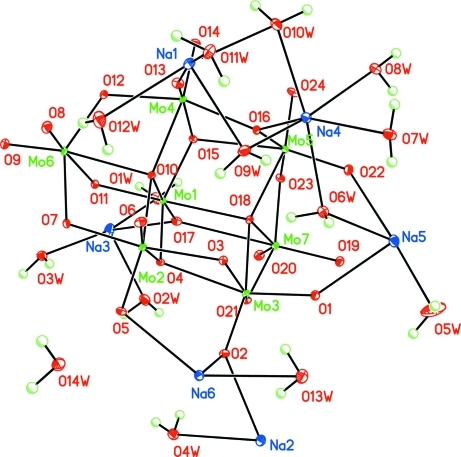
The molecular structure of (I) with displacement ellipsoids are drawn at the 30% probability level; H atoms are given as spheres of arbitrary radius.

**Fig. 2.**
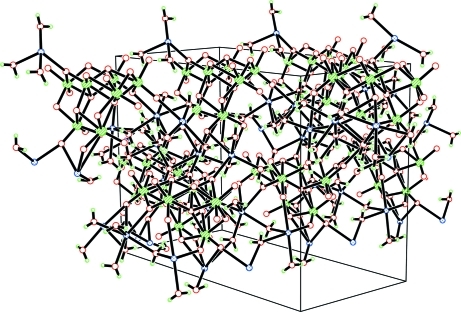
The crystal packing of (I), displayed with O—H···O hydrogen bonds as dashed lines.

## supplementary crystallographic information

### Comment

The design and synthesis of polyoxometalates has attracted continuous research interest not only because of their appealing structural and topological novelties, but also due to their interesting optical, electronic, magnetic, and catalytic properties, as well as their potential medical applications (Pope & Müller, 1991). In our research group, a series of polyoxomolybdate structures have been reported (Zhang, Dou *et al.*, 2009; Zhang, Wei *et al.*, 2009). Here, we describe the synthesis and structural characterization of the title compound.

As shown in Figure 1, consists of six sodium cations, one Mo_7_O_24_ anion, and fourteen water molecules. The Na^+^ cations are in a distorted octahedral environment, coordinated by six neighboring water molecules. Na—O bond lengths are in the normal range of 2.331 (4)—2.692 (4) Å, compared to the reported ones (Turpeinen *et al.*, 2001; An *et al.*, 2004).

The configuration of the heptamolybdate anion consisting of seven edge-sharing MoO_6_ octahedra is very similar to that reported for other heptamolybdates (Evans *et al.*, 1975; Yang *et al.*, 2002). The X-ray analysis shows the arrangement in terms of polyhedra, in which three octahedra are approximately in line in the central horizontal level and four are attached forward at a level above. In each heptamolybdate anion, six peripheral Mo atoms (Mo2, Mo3, Mo4 Mo5, Mo6 and Mo7) have two terminal oxygens (t—O), two bridging oxygens (β—O), one capping oxygen (β3—O), and one β4—O atom bonded to four Mo atoms, while the seventh Mo center (Mo1) has four capping oxygens and two β4—O atoms. Although Mo1 has no terminal oxygen atom, Mo=O characters are still obvious in those two very short Mo—O distances [Mo1—O11, 1.746 (3) Å and Mo1—O17, 1.727 (3) Å) opposed to two abnormally long Mo—O distances [Mo1—O10, 2.298 (3) Å and Mo1—O18, 2.238 (3) Å).

O—H···O hydrogen bonding between anionic moieties and water molecules leads to a consolidation of the structure (Fig. 2; Table 2).

### Experimental

A mixture of 2,4'-biphenyldicarboxylic acid (0.2 mmoL 0.05 g), 2-Pyridyl)pyrazole (0.3 mmoL 0.05 g), sodium molybdate (0.4 mmoL, 0.10 g), and copper(II) sulfate pentahydrate (0.2 mmol, 0.05 g) in 14 ml distilled water was sealed in a 25 ml Teflon-lined stainless steel autoclave and was kept at 433 K for three days. Colourless blocks of (I) were obtained.

### Refinement

The water H atoms were located in difference maps and refined by using the 'DFIX' command with H···H = 1.38 Å, and O—H = 0.82 (2) Å and U_iso_ = 1.5U_eq_(O).

### Crystal data

**Table:**

Na_6_(Mo_7_O_24_)·14H_2_O	*F*(000) = 2768
*M**_r_* = 1445.74	*D*_x_ = 2.807 Mg m^−^^3^
Orthorhombic, *P**c**a*2_1_	Mo *K*α radiation, λ = 0.71073 Å
Hall symbol: P 2c -2ac	Cell parameters from 9872 reflections
*a* = 21.1304 (2) Å	θ = 2.2–30.3°
*b* = 10.3733 (1) Å	µ = 2.68 mm^−^^1^
*c* = 15.6094 (2) Å	*T* = 296 K
*V* = 3421.46 (6) Å^3^	Block, colourless
*Z* = 4	0.12 × 0.10 × 0.08 mm

### Data collection

**Table:**

Bruker APEXII CCD diffractometer	5831 independent reflections
Radiation source: fine-focus sealed tube	5748 reflections with *I* > 2σ(*I*)
graphite	*R*_int_ = 0.021
ω scans	θ_max_ = 25.0°, θ_min_ = 1.9°
Absorption correction: multi-scan (*SADABS*; Bruker, 2001)	*h* = −20→25
*T*_min_ = 0.739, *T*_max_ = 0.814	*k* = −12→11
16564 measured reflections	*l* = −18→18

### Refinement

**Table:**

Refinement on *F*^2^	Hydrogen site location: difference Fourier map
Least-squares matrix: full	H atoms treated by a mixture of independent and constrained refinement
*R*[*F*^2^ > 2σ(*F*^2^)] = 0.017	*w* = 1/[σ^2^(*F*_o_^2^) + (0.029*P*)^2^] where *P* = (*F*_o_^2^ + 2*F*_c_^2^)/3
*wR*(*F*^2^) = 0.044	(Δ/σ)_max_ = 0.001
*S* = 1.00	Δρ_max_ = 1.12 e Å^−^^3^
5831 reflections	Δρ_min_ = −1.00 e Å^−^^3^
545 parameters	Extinction correction: *SHELXL*, Fc^*^=kFc[1+0.001xFc^2^λ^3^/sin(2θ)]^-1/4^
63 restraints	Extinction coefficient: 0.00258 (7)
Primary atom site location: structure-invariant direct methods	Absolute structure: Flack (1983), 2689 Friedel pairs
Secondary atom site location: difference Fourier map	Flack parameter: −0.02 (2)

### Special details

**Table:**

Geometry. All esds (except the esd in the dihedral angle between two l.s. planes) are estimated using the full covariance matrix. The cell esds are taken into account individually in the estimation of esds in distances, angles and torsion angles; correlations between esds in cell parameters are only used when they are defined by crystal symmetry. An approximate (isotropic) treatment of cell esds is used for estimating esds involving l.s. planes.
Refinement. Refinement of *F*^2^ against ALL reflections. The weighted *R*-factor wR and goodness of fit *S* are based on *F*^2^, conventional *R*-factors *R* are based on *F*, with *F* set to zero for negative *F*^2^. The threshold expression of *F*^2^ > σ(*F*^2^) is used only for calculating *R*-factors(gt) etc. and is not relevant to the choice of reflections for refinement. *R*-factors based on *F*^2^ are statistically about twice as large as those based on *F*, and *R*- factors based on ALL data will be even larger.

### Fractional atomic coordinates and isotropic or equivalent isotropic displacement parameters (Å^2^)

**Table:**

	*x*	*y*	*z*	*U*_iso_*/*U*_eq_	
Mo1	0.441554 (16)	0.17102 (3)	0.95800 (3)	0.01241 (9)	
Mo2	0.519892 (17)	0.17010 (4)	0.76582 (3)	0.01523 (10)	
Mo3	0.364901 (18)	0.18576 (4)	0.76633 (3)	0.01518 (10)	
Mo4	0.528881 (18)	0.44744 (4)	0.95766 (3)	0.01502 (9)	
Mo5	0.374213 (18)	0.47162 (4)	0.95430 (3)	0.01652 (10)	
Mo6	0.603656 (18)	0.18041 (4)	0.93595 (3)	0.01647 (10)	
Mo7	0.282633 (18)	0.22516 (4)	0.93663 (3)	0.01714 (10)	
Na1	0.64293 (9)	0.6312 (2)	0.78932 (12)	0.0257 (4)	
Na2	0.27899 (10)	−0.0513 (2)	0.60777 (14)	0.0311 (5)	
Na3	0.38753 (11)	−0.0891 (3)	1.10058 (16)	0.0458 (7)	
Na4	0.48165 (9)	0.66440 (19)	0.79018 (13)	0.0260 (5)	
Na5	0.30429 (10)	0.5433 (2)	0.71744 (16)	0.0364 (6)	
Na6	0.44153 (9)	0.0751 (2)	0.57623 (13)	0.0262 (5)	
O1	0.31809 (17)	0.3039 (4)	0.7229 (2)	0.0243 (8)	
O2	0.36309 (17)	0.0599 (3)	0.6943 (2)	0.0241 (8)	
O3	0.44497 (14)	0.2636 (3)	0.7335 (2)	0.0175 (7)	
O4	0.43894 (15)	0.0915 (3)	0.8473 (2)	0.0155 (7)	
O5	0.51294 (17)	0.0389 (3)	0.6968 (2)	0.0233 (8)	
O6	0.57476 (17)	0.2655 (4)	0.7163 (2)	0.0253 (8)	
O7	0.57567 (17)	0.0718 (3)	0.8441 (2)	0.0206 (7)	
O8	0.66719 (17)	0.2509 (4)	0.8854 (3)	0.0301 (9)	
O9	0.63533 (17)	0.0697 (3)	1.0055 (2)	0.0255 (8)	
O10	0.52100 (14)	0.2723 (3)	0.8833 (2)	0.0150 (7)	
O11	0.50591 (16)	0.0893 (3)	1.0023 (2)	0.0210 (7)	
O12	0.58294 (17)	0.3174 (3)	1.0138 (2)	0.0194 (7)	
O13	0.58341 (16)	0.5165 (4)	0.8912 (2)	0.0266 (8)	
O14	0.52789 (18)	0.5444 (3)	1.0477 (2)	0.0259 (8)	
O15	0.44765 (14)	0.3318 (3)	1.0129 (2)	0.0153 (7)	
O16	0.45439 (14)	0.5087 (3)	0.9006 (2)	0.0184 (7)	
O17	0.37682 (17)	0.1012 (3)	1.0071 (2)	0.0238 (8)	
O18	0.37170 (14)	0.2913 (3)	0.8856 (2)	0.0156 (7)	
O19	0.22488 (17)	0.3105 (4)	0.8851 (2)	0.0299 (9)	
O20	0.24370 (17)	0.1265 (4)	1.0080 (2)	0.0269 (8)	
O21	0.30435 (16)	0.1066 (3)	0.8458 (2)	0.0183 (7)	
O22	0.32638 (17)	0.5509 (4)	0.8837 (2)	0.0290 (9)	
O23	0.31128 (15)	0.3577 (3)	1.0128 (2)	0.0195 (7)	
O24	0.37878 (19)	0.5698 (4)	1.0429 (3)	0.0307 (9)	
O1W	0.3174 (2)	−0.0027 (5)	1.1998 (3)	0.0462 (11)	
O2W	0.2972 (3)	−0.1913 (5)	1.0428 (3)	0.0478 (12)	
O3W	0.4691 (2)	−0.1981 (4)	1.0305 (2)	0.0296 (9)	
O4W	0.3203 (2)	−0.2267 (4)	0.6941 (3)	0.0352 (9)	
O5W	0.2912 (3)	0.5327 (7)	0.5640 (4)	0.086 (2)	
O6W	0.41466 (18)	0.5240 (4)	0.7083 (2)	0.0288 (8)	
O7W	0.4549 (2)	0.7974 (4)	0.6737 (3)	0.0344 (10)	
O8W	0.4210 (2)	0.8248 (4)	0.8668 (3)	0.0302 (9)	
O9W	0.5612 (2)	0.5692 (5)	0.6945 (3)	0.0376 (10)	
O10W	0.5724 (2)	0.7960 (4)	0.8444 (3)	0.0366 (10)	
O11W	0.69267 (19)	0.7408 (4)	0.6777 (2)	0.0323 (9)	
O12W	0.6942 (2)	0.4283 (4)	0.7449 (3)	0.0491 (12)	
O13W	0.38065 (18)	0.2485 (3)	0.5149 (3)	0.0421 (10)	
O14W	0.71393 (19)	0.1461 (4)	0.3624 (3)	0.0348 (10)	
H1W	0.316 (3)	0.0761 (6)	1.196 (4)	0.080*	
H2W	0.326 (3)	−0.029 (5)	1.2479 (15)	0.080*	
H3W	0.295 (3)	−0.167 (5)	0.9929 (12)	0.080*	
H4W	0.299 (4)	−0.2700 (9)	1.049 (3)	0.080*	
H5W	0.478 (3)	−0.170 (4)	0.982 (2)	0.080*	
H6W	0.467 (4)	−0.2784 (7)	1.034 (4)	0.080*	
H7W	0.306 (2)	−0.208 (6)	0.7412 (14)	0.080*	
H8W	0.3579 (12)	−0.209 (8)	0.688 (4)	0.080*	
H9W	0.287 (3)	0.4546 (11)	0.559 (4)	0.080*	
H10W	0.318 (3)	0.565 (5)	0.532 (4)	0.080*	
H11W	0.428 (2)	0.537 (5)	0.6599 (12)	0.080*	
H12W	0.427 (2)	0.456 (3)	0.730 (3)	0.080*	
H13W	0.455 (3)	0.768 (5)	0.6248 (14)	0.080*	
H14W	0.477 (3)	0.862 (5)	0.680 (4)	0.080*	
H15W	0.3839 (8)	0.803 (5)	0.861 (5)	0.080*	
H16W	0.430 (3)	0.895 (4)	0.845 (6)	0.080*	
H17W	0.562 (4)	0.4902 (5)	0.693 (4)	0.080*	
H18W	0.559 (3)	0.603 (5)	0.6473 (17)	0.080*	
H19W	0.584 (3)	0.784 (6)	0.8939 (13)	0.080*	
H20W	0.564 (3)	0.8712 (15)	0.832 (4)	0.080*	
H21W	0.7309 (5)	0.731 (7)	0.683 (4)	0.080*	
H22W	0.679 (3)	0.726 (9)	0.629 (2)	0.080*	
H23W	0.687 (3)	0.376 (4)	0.783 (3)	0.080*	
H24W	0.684 (4)	0.405 (6)	0.6968 (18)	0.080*	
H25W	0.374 (3)	0.172 (2)	0.526 (5)	0.080*	
H26W	0.413 (2)	0.278 (5)	0.535 (5)	0.080*	
H27W	0.716 (3)	0.0671 (5)	0.361 (6)	0.080*	
H28W	0.7484 (13)	0.182 (5)	0.361 (6)	0.080*	

### Atomic displacement parameters (Å^2^)

**Table:**

	*U*^11^	*U*^22^	*U*^33^	*U*^12^	*U*^13^	*U*^23^
Mo1	0.0120 (2)	0.01206 (19)	0.0131 (2)	−0.00056 (13)	−0.00043 (16)	0.00097 (16)
Mo2	0.0147 (2)	0.0172 (2)	0.01380 (19)	−0.00019 (14)	0.00116 (17)	−0.00129 (16)
Mo3	0.0143 (2)	0.0164 (2)	0.01480 (19)	−0.00104 (15)	−0.00192 (17)	−0.00082 (16)
Mo4	0.01592 (19)	0.01323 (19)	0.01592 (19)	−0.00242 (14)	−0.00039 (17)	0.00063 (16)
Mo5	0.01692 (19)	0.01323 (19)	0.0194 (2)	0.00094 (14)	0.00030 (17)	−0.00124 (16)
Mo6	0.01212 (19)	0.0185 (2)	0.0187 (2)	0.00150 (14)	−0.00171 (16)	0.00215 (16)
Mo7	0.01230 (19)	0.0199 (2)	0.0192 (2)	−0.00179 (15)	0.00169 (16)	0.00027 (17)
Na1	0.0219 (10)	0.0308 (11)	0.0244 (11)	−0.0043 (8)	0.0011 (8)	0.0013 (9)
Na2	0.0243 (11)	0.0370 (13)	0.0321 (11)	0.0014 (9)	0.0002 (9)	0.0022 (10)
Na3	0.0321 (13)	0.0686 (19)	0.0366 (13)	0.0158 (12)	0.0011 (10)	0.0072 (13)
Na4	0.0311 (12)	0.0249 (11)	0.0219 (10)	−0.0007 (8)	0.0007 (8)	0.0024 (8)
Na5	0.0246 (12)	0.0302 (13)	0.0543 (15)	0.0009 (9)	0.0001 (10)	0.0007 (11)
Na6	0.0241 (11)	0.0284 (12)	0.0260 (11)	0.0030 (8)	−0.0005 (8)	−0.0041 (9)
O1	0.0218 (19)	0.025 (2)	0.027 (2)	0.0010 (15)	−0.0034 (15)	0.0026 (15)
O2	0.0251 (19)	0.0249 (19)	0.0223 (19)	−0.0018 (15)	−0.0007 (15)	−0.0037 (15)
O3	0.0144 (17)	0.0182 (18)	0.0198 (18)	−0.0009 (13)	0.0017 (12)	0.0042 (14)
O4	0.0175 (18)	0.0149 (17)	0.0140 (17)	−0.0016 (13)	0.0002 (12)	−0.0010 (13)
O5	0.026 (2)	0.022 (2)	0.0210 (18)	0.0011 (14)	−0.0012 (15)	−0.0060 (15)
O6	0.0202 (18)	0.029 (2)	0.026 (2)	−0.0086 (15)	0.0028 (15)	0.0000 (15)
O7	0.0232 (19)	0.0191 (18)	0.0196 (18)	0.0089 (15)	−0.0032 (14)	−0.0017 (14)
O8	0.0200 (19)	0.032 (2)	0.038 (2)	−0.0013 (16)	0.0051 (17)	0.0019 (17)
O9	0.0207 (19)	0.027 (2)	0.0284 (19)	0.0035 (15)	−0.0036 (15)	0.0043 (16)
O10	0.0129 (17)	0.0169 (18)	0.0152 (16)	−0.0006 (12)	−0.0004 (13)	−0.0015 (13)
O11	0.0217 (18)	0.0199 (18)	0.0215 (17)	0.0038 (14)	−0.0044 (15)	0.0019 (14)
O12	0.0197 (17)	0.0209 (18)	0.0178 (18)	0.0015 (13)	−0.0056 (15)	0.0011 (14)
O13	0.025 (2)	0.028 (2)	0.027 (2)	−0.0051 (15)	0.0063 (15)	0.0025 (16)
O14	0.034 (2)	0.022 (2)	0.0216 (19)	0.0002 (15)	−0.0038 (16)	−0.0059 (15)
O15	0.0169 (17)	0.0159 (17)	0.0131 (17)	−0.0004 (12)	0.0025 (13)	−0.0002 (13)
O16	0.0196 (17)	0.0150 (17)	0.0205 (17)	−0.0006 (13)	−0.0024 (13)	0.0029 (14)
O17	0.0273 (19)	0.0213 (18)	0.0229 (18)	−0.0062 (14)	0.0036 (15)	0.0020 (14)
O18	0.0124 (16)	0.0198 (17)	0.0146 (17)	−0.0009 (13)	0.0017 (13)	0.0010 (14)
O19	0.0235 (19)	0.034 (2)	0.032 (2)	0.0050 (16)	−0.0054 (17)	−0.0014 (17)
O20	0.0202 (19)	0.031 (2)	0.0292 (19)	−0.0080 (16)	0.0041 (15)	0.0039 (17)
O21	0.0182 (18)	0.0191 (18)	0.0176 (17)	−0.0076 (14)	−0.0002 (14)	−0.0005 (13)
O22	0.025 (2)	0.027 (2)	0.035 (2)	0.0029 (16)	−0.0056 (17)	0.0051 (17)
O23	0.0197 (17)	0.0194 (17)	0.0192 (17)	−0.0010 (14)	0.0058 (14)	−0.0020 (14)
O24	0.039 (2)	0.0199 (19)	0.033 (2)	−0.0003 (16)	0.0012 (18)	−0.0086 (16)
O1W	0.051 (3)	0.042 (3)	0.046 (3)	−0.001 (2)	0.006 (2)	−0.003 (2)
O2W	0.066 (3)	0.040 (3)	0.038 (3)	0.014 (2)	−0.004 (2)	−0.006 (2)
O3W	0.032 (2)	0.027 (2)	0.030 (2)	−0.0028 (17)	−0.0004 (18)	0.0008 (16)
O4W	0.036 (2)	0.030 (2)	0.039 (2)	0.0001 (18)	−0.0006 (19)	−0.0010 (19)
O5W	0.096 (5)	0.107 (5)	0.054 (3)	−0.064 (4)	−0.009 (3)	0.002 (3)
O6W	0.029 (2)	0.025 (2)	0.032 (2)	−0.0035 (16)	0.0051 (17)	0.0035 (16)
O7W	0.053 (3)	0.025 (2)	0.025 (2)	−0.0062 (19)	0.0011 (18)	0.0016 (17)
O8W	0.034 (2)	0.026 (2)	0.031 (2)	−0.0006 (17)	0.0015 (19)	−0.0023 (16)
O9W	0.030 (2)	0.047 (3)	0.036 (2)	0.002 (2)	−0.0058 (18)	−0.015 (2)
O10W	0.046 (3)	0.023 (2)	0.041 (3)	0.0031 (19)	−0.004 (2)	−0.0026 (18)
O11W	0.024 (2)	0.046 (3)	0.026 (2)	−0.0014 (18)	−0.0036 (16)	0.0077 (18)
O12W	0.034 (2)	0.041 (3)	0.073 (3)	0.0010 (19)	0.013 (2)	0.006 (2)
O13W	0.035 (2)	0.041 (3)	0.051 (3)	−0.0027 (19)	−0.008 (2)	0.010 (2)
O14W	0.026 (2)	0.031 (2)	0.047 (2)	−0.0036 (18)	−0.0036 (19)	−0.005 (2)

### Geometric parameters (Å, °)

**Table:**

Mo1—O17	1.727 (3)	Na4—O7W	2.352 (5)
Mo1—O11	1.746 (3)	Na4—O6W	2.399 (4)
Mo1—O15	1.880 (3)	Na4—O8W	2.417 (5)
Mo1—O4	1.915 (3)	Na4—O16	2.430 (4)
Mo1—O18	2.238 (3)	Na4—O9W	2.455 (5)
Mo1—O10	2.298 (3)	Na4—O10W	2.501 (5)
Mo2—O6	1.709 (3)	Na5—O6W	2.345 (4)
Mo2—O5	1.741 (3)	Na5—O12W^v^	2.384 (5)
Mo2—O3	1.924 (3)	Na5—O5W	2.413 (7)
Mo2—O7	1.981 (3)	Na5—O4W^vi^	2.437 (5)
Mo2—O10	2.119 (3)	Na5—O1	2.503 (4)
Mo2—O4	2.283 (3)	Na5—O22	2.638 (5)
Mo3—O1	1.715 (4)	Na6—O13W	2.410 (4)
Mo3—O2	1.723 (3)	Na6—O11^iii^	2.339 (4)
Mo3—O3	1.943 (3)	Na6—O3W^iii^	2.388 (5)
Mo3—O21	1.962 (3)	Na6—O5	2.442 (4)
Mo3—O18	2.164 (3)	Na6—O9^iii^	2.472 (4)
Mo3—O4	2.237 (3)	Na6—O2	2.484 (4)
Mo4—O13	1.707 (3)	O5—Na3^iii^	2.637 (4)
Mo4—O14	1.729 (3)	O6—Na3^iii^	2.692 (4)
Mo4—O16	1.917 (3)	O9—Na2^iv^	2.421 (4)
Mo4—O12	1.973 (3)	O9—Na6^iv^	2.472 (4)
Mo4—O10	2.162 (3)	O11—Na6^iv^	2.339 (4)
Mo4—O15	2.264 (3)	O19—Na1^v^	2.366 (4)
Mo5—O22	1.706 (4)	O20—Na2^vii^	2.461 (4)
Mo5—O24	1.720 (4)	O1W—Na2^vii^	2.543 (5)
Mo5—O16	1.929 (3)	O12W—Na5^i^	2.384 (5)
Mo5—O23	2.000 (3)	O1W—H1W	0.821 (4)
Mo5—O18	2.157 (3)	O1W—H2W	0.82 (3)
Mo5—O15	2.312 (3)	O2W—Na2^vii^	2.395 (5)
Mo6—O9	1.717 (3)	O2W—H3W	0.82 (3)
Mo6—O8	1.720 (4)	O2W—H4W	0.823 (15)
Mo6—O7	1.917 (3)	O3W—Na6^iv^	2.388 (5)
Mo6—O12	1.920 (3)	O3W—H5W	0.83 (4)
Mo6—O10	2.153 (3)	O3W—H6W	0.835 (9)
Mo6—O11	2.497 (4)	O4W—Na5^viii^	2.437 (5)
Mo7—O19	1.709 (4)	O4W—H7W	0.82 (3)
Mo7—O20	1.722 (4)	O4W—H8W	0.82 (3)
Mo7—O23	1.915 (3)	O5W—H9W	0.819 (15)
Mo7—O21	1.932 (3)	O5W—H10W	0.83 (6)
Mo7—O18	2.156 (3)	O6W—H11W	0.82 (3)
Na1—O11W	2.331 (4)	O6W—H12W	0.83 (4)
Na1—O13	2.352 (4)	O7W—H13W	0.82 (3)
Na1—O9W	2.364 (5)	O7W—H14W	0.82 (6)
Na1—O19^i^	2.366 (4)	O8W—H15W	0.82 (2)
Na1—O10W	2.426 (5)	O8W—H16W	0.83 (5)
Na1—O12W	2.467 (5)	O9W—H17W	0.820 (10)
Na2—O2W^ii^	2.395 (5)	O9W—H18W	0.82 (3)
Na2—O9^iii^	2.421 (4)	O10W—H19W	0.82 (3)
Na2—O4W	2.427 (5)	O10W—H20W	0.82 (2)
Na2—O20^ii^	2.461 (4)	O11W—H21W	0.818 (15)
Na2—O2	2.513 (4)	O11W—H22W	0.83 (4)
Na2—O1W^ii^	2.543 (5)	O12W—H23W	0.82 (5)
Na3—O1W	2.324 (5)	O12W—H24W	0.82 (4)
Na3—O3W	2.334 (5)	O13W—H25W	0.82 (3)
Na3—O2W	2.362 (6)	O13W—H26W	0.81 (5)
Na3—O17	2.466 (4)	O14W—H27W	0.821 (9)
Na3—O5^iv^	2.637 (4)	O14W—H28W	0.82 (4)
Na3—O6^iv^	2.692 (4)		
			
O17—Mo1—O11	103.74 (18)	O2W^ii^—Na2—O1W^ii^	79.69 (18)
O17—Mo1—O15	102.93 (16)	O9^iii^—Na2—O1W^ii^	170.76 (17)
O11—Mo1—O15	101.36 (15)	O4W—Na2—O1W^ii^	97.07 (17)
O17—Mo1—O4	101.36 (15)	O20^ii^—Na2—O1W^ii^	93.04 (16)
O11—Mo1—O4	99.83 (15)	O2—Na2—O1W^ii^	99.87 (15)
O15—Mo1—O4	142.70 (15)	O1W—Na3—O3W	166.03 (19)
O17—Mo1—O18	86.29 (15)	O1W—Na3—O2W	84.99 (18)
O11—Mo1—O18	169.87 (14)	O3W—Na3—O2W	101.55 (18)
O15—Mo1—O18	77.34 (13)	O1W—Na3—O17	91.55 (17)
O4—Mo1—O18	76.45 (13)	O3W—Na3—O17	100.28 (16)
O17—Mo1—O10	174.48 (15)	O2W—Na3—O17	93.40 (17)
O11—Mo1—O10	81.61 (14)	O1W—Na3—O5^iv^	93.01 (16)
O15—Mo1—O10	77.03 (13)	O3W—Na3—O5^iv^	76.91 (14)
O4—Mo1—O10	76.14 (13)	O2W—Na3—O5^iv^	161.95 (19)
O18—Mo1—O10	88.33 (12)	O17—Na3—O5^iv^	104.60 (15)
O6—Mo2—O5	103.31 (17)	O1W—Na3—O6^iv^	90.19 (17)
O6—Mo2—O3	98.51 (16)	O3W—Na3—O6^iv^	76.51 (14)
O5—Mo2—O3	99.36 (16)	O2W—Na3—O6^iv^	100.99 (18)
O6—Mo2—O7	99.99 (17)	O17—Na3—O6^iv^	165.60 (16)
O5—Mo2—O7	91.69 (16)	O5^iv^—Na3—O6^iv^	61.03 (12)
O3—Mo2—O7	155.63 (14)	O7W—Na4—O6W	78.62 (15)
O6—Mo2—O10	95.41 (15)	O7W—Na4—O8W	81.45 (16)
O5—Mo2—O10	158.01 (15)	O6W—Na4—O8W	111.64 (16)
O3—Mo2—O10	89.05 (13)	O7W—Na4—O16	151.77 (16)
O7—Mo2—O10	73.56 (13)	O6W—Na4—O16	80.47 (13)
O6—Mo2—O4	165.32 (15)	O8W—Na4—O16	88.92 (14)
O5—Mo2—O4	90.13 (14)	O7W—Na4—O9W	85.99 (17)
O3—Mo2—O4	73.11 (13)	O6W—Na4—O9W	80.55 (14)
O7—Mo2—O4	85.29 (13)	O8W—Na4—O9W	160.16 (18)
O10—Mo2—O4	72.81 (12)	O16—Na4—O9W	109.03 (16)
O1—Mo3—O2	105.69 (17)	O7W—Na4—O10W	97.22 (17)
O1—Mo3—O3	95.81 (16)	O6W—Na4—O10W	164.52 (16)
O2—Mo3—O3	99.35 (16)	O8W—Na4—O10W	82.12 (15)
O1—Mo3—O21	99.95 (16)	O16—Na4—O10W	107.71 (15)
O2—Mo3—O21	94.63 (16)	O9W—Na4—O10W	84.31 (16)
O3—Mo3—O21	155.25 (14)	O6W—Na5—O12W^v^	172.8 (2)
O1—Mo3—O18	91.01 (15)	O6W—Na5—O5W	92.9 (2)
O2—Mo3—O18	161.01 (15)	O12W^v^—Na5—O5W	94.1 (2)
O3—Mo3—O18	87.63 (13)	O6W—Na5—O4W^vi^	86.34 (15)
O21—Mo3—O18	73.21 (12)	O12W^v^—Na5—O4W^vi^	92.41 (17)
O1—Mo3—O4	160.00 (15)	O5W—Na5—O4W^vi^	85.0 (2)
O2—Mo3—O4	93.03 (15)	O6W—Na5—O1	78.54 (14)
O3—Mo3—O4	73.83 (13)	O12W^v^—Na5—O1	103.31 (16)
O21—Mo3—O4	85.18 (13)	O5W—Na5—O1	90.1 (2)
O18—Mo3—O4	71.84 (12)	O4W^vi^—Na5—O1	163.84 (16)
O13—Mo4—O14	104.96 (18)	O6W—Na5—O22	83.46 (14)
O13—Mo4—O16	97.62 (16)	O12W^v^—Na5—O22	89.57 (18)
O14—Mo4—O16	100.09 (16)	O5W—Na5—O22	176.3 (2)
O13—Mo4—O12	99.54 (16)	O4W^vi^—Na5—O22	95.37 (15)
O14—Mo4—O12	92.49 (16)	O1—Na5—O22	88.60 (13)
O16—Mo4—O12	155.31 (14)	O13W—Na6—O11^iii^	126.98 (17)
O13—Mo4—O10	94.50 (15)	O13W—Na6—O3W^iii^	84.49 (15)
O14—Mo4—O10	157.58 (15)	O11^iii^—Na6—O3W^iii^	82.35 (14)
O16—Mo4—O10	88.05 (13)	O13W—Na6—O5	138.66 (17)
O12—Mo4—O10	73.06 (13)	O11^iii^—Na6—O5	88.57 (13)
O13—Mo4—O15	164.76 (15)	O3W^iii^—Na6—O5	79.87 (14)
O14—Mo4—O15	89.38 (15)	O13W—Na6—O9^iii^	85.71 (15)
O16—Mo4—O15	74.36 (13)	O11^iii^—Na6—O9^iii^	69.45 (13)
O12—Mo4—O15	84.70 (13)	O3W^iii^—Na6—O9^iii^	135.27 (15)
O10—Mo4—O15	72.60 (12)	O5—Na6—O9^iii^	131.09 (15)
O22—Mo5—O24	105.50 (19)	O13W—Na6—O2	89.20 (15)
O22—Mo5—O16	98.23 (16)	O11^iii^—Na6—O2	129.55 (15)
O24—Mo5—O16	100.49 (17)	O3W^iii^—Na6—O2	141.55 (15)
O22—Mo5—O23	100.70 (16)	O5—Na6—O2	80.23 (14)
O24—Mo5—O23	91.18 (16)	O9^iii^—Na6—O2	81.64 (13)
O16—Mo5—O23	154.21 (14)	Mo3—O1—Na5	142.2 (2)
O22—Mo5—O18	94.71 (15)	Mo3—O2—Na6	114.91 (18)
O24—Mo5—O18	156.14 (16)	Mo3—O2—Na2	135.58 (19)
O16—Mo5—O18	88.77 (13)	Na6—O2—Na2	95.85 (14)
O23—Mo5—O18	72.41 (13)	Mo2—O3—Mo3	115.98 (17)
O22—Mo5—O15	162.75 (16)	Mo1—O4—Mo3	109.98 (15)
O24—Mo5—O15	90.90 (16)	Mo1—O4—Mo2	109.10 (14)
O16—Mo5—O15	73.01 (12)	Mo3—O4—Mo2	93.03 (12)
O23—Mo5—O15	83.97 (13)	Mo2—O5—Na6	114.14 (18)
O18—Mo5—O15	70.68 (12)	Mo2—O5—Na3^iii^	97.50 (16)
O9—Mo6—O8	105.68 (18)	Na6—O5—Na3^iii^	91.33 (14)
O9—Mo6—O7	101.52 (16)	Mo2—O6—Na3^iii^	96.37 (17)
O8—Mo6—O7	98.49 (17)	Mo6—O7—Mo2	110.01 (17)
O9—Mo6—O12	100.59 (16)	Mo6—O9—Na2^iv^	139.6 (2)
O8—Mo6—O12	98.84 (17)	Mo6—O9—Na6^iv^	115.65 (18)
O7—Mo6—O12	146.93 (15)	Na2^iv^—O9—Na6^iv^	98.57 (14)
O9—Mo6—O10	148.66 (15)	Mo2—O10—Mo6	96.77 (13)
O8—Mo6—O10	105.66 (16)	Mo2—O10—Mo4	152.37 (17)
O7—Mo6—O10	74.02 (13)	Mo6—O10—Mo4	96.01 (12)
O12—Mo6—O10	74.28 (13)	Mo2—O10—Mo1	101.67 (13)
O9—Mo6—O11	78.85 (14)	Mo6—O10—Mo1	101.34 (13)
O8—Mo6—O11	175.47 (15)	Mo4—O10—Mo1	99.68 (12)
O7—Mo6—O11	80.35 (13)	Mo1—O11—Na6^iv^	156.9 (2)
O12—Mo6—O11	80.15 (13)	Mo1—O11—Mo6	107.22 (16)
O10—Mo6—O11	69.81 (11)	Na6^iv^—O11—Mo6	95.04 (13)
O19—Mo7—O20	105.74 (19)	Mo6—O12—Mo4	110.89 (16)
O19—Mo7—O23	98.41 (17)	Mo4—O13—Na1	169.7 (2)
O20—Mo7—O23	100.14 (17)	Mo1—O15—Mo4	110.38 (15)
O19—Mo7—O21	98.83 (17)	Mo1—O15—Mo5	109.28 (15)
O20—Mo7—O21	102.13 (16)	Mo4—O15—Mo5	91.48 (12)
O23—Mo7—O21	146.83 (14)	Mo4—O16—Mo5	116.91 (17)
O19—Mo7—O18	106.55 (16)	Mo4—O16—Na4	110.82 (15)
O20—Mo7—O18	147.69 (15)	Mo5—O16—Na4	130.46 (16)
O23—Mo7—O18	74.04 (13)	Mo1—O17—Na3	121.71 (19)
O21—Mo7—O18	73.97 (13)	Mo7—O18—Mo5	96.53 (12)
O11W—Na1—O13	173.62 (16)	Mo7—O18—Mo3	95.68 (12)
O11W—Na1—O9W	89.69 (16)	Mo5—O18—Mo3	150.20 (16)
O13—Na1—O9W	83.96 (16)	Mo7—O18—Mo1	102.24 (13)
O11W—Na1—O19^i^	91.00 (15)	Mo5—O18—Mo1	102.48 (13)
O13—Na1—O19^i^	95.36 (15)	Mo3—O18—Mo1	101.32 (13)
O9W—Na1—O19^i^	178.99 (19)	Mo7—O19—Na1^v^	162.3 (2)
O11W—Na1—O10W	101.45 (18)	Mo7—O20—Na2^vii^	161.4 (2)
O13—Na1—O10W	77.76 (15)	Mo7—O21—Mo3	110.65 (16)
O9W—Na1—O10W	87.96 (16)	Mo5—O22—Na5	136.5 (2)
O19^i^—Na1—O10W	92.63 (16)	Mo7—O23—Mo5	110.56 (15)
O11W—Na1—O12W	90.47 (17)	H1W—O1W—H2W	114 (6)
O13—Na1—O12W	89.60 (16)	H3W—O2W—H4W	115 (5)
O9W—Na1—O12W	84.97 (18)	H5W—O3W—H6W	115 (5)
O19^i^—Na1—O12W	94.28 (17)	H7W—O4W—H8W	114 (6)
O10W—Na1—O12W	166.11 (18)	H9W—O5W—H10W	115 (6)
O2W^ii^—Na2—O9^iii^	100.14 (17)	H11W—O6W—H12W	114 (5)
O2W^ii^—Na2—O4W	91.30 (17)	H13W—O7W—H14W	114 (6)
O9^iii^—Na2—O4W	92.17 (15)	H15W—O8W—H16W	115 (6)
O2W^ii^—Na2—O20^ii^	93.17 (17)	H17W—O9W—H18W	114 (5)
O9^iii^—Na2—O20^ii^	77.72 (14)	H19W—O10W—H20W	116 (6)
O4W—Na2—O20^ii^	169.53 (16)	H21W—O11W—H22W	115 (6)
O2W^ii^—Na2—O2	169.12 (18)	H23W—O12W—H24W	115 (5)
O9^iii^—Na2—O2	82.06 (13)	H25W—O13W—H26W	115 (6)
O4W—Na2—O2	77.93 (14)	H27W—O14W—H28W	114 (5)
O20^ii^—Na2—O2	97.71 (14)		

Symmetry codes: (i) *x*+1/2, −*y*+1, *z*; (ii) −*x*+1/2, *y*, *z*−1/2; (iii) −*x*+1, −*y*, *z*−1/2; (iv) −*x*+1, −*y*, *z*+1/2; (v) *x*−1/2, −*y*+1, *z*; (vi) *x*, *y*+1, *z*; (vii) −*x*+1/2, *y*, *z*+1/2; (viii) *x*, *y*−1, *z*.

### Hydrogen-bond geometry (Å, °)

**Table:**

*D*—H···*A*	*D*—H	H···*A*	*D*···*A*	*D*—H···*A*
O6W—H12W···O3	0.83 (4)	2.04 (2)	2.804 (5)	156 (5)
O9W—H17W···O6	0.82 (1)	2.37 (1)	3.182 (6)	168 (6)
O12W—H23W···O8	0.82 (5)	2.10 (5)	2.920 (6)	176 (5)
O12W—H24W···O6	0.82 (4)	2.73 (7)	3.069 (6)	106 (6)
O1W—H1W···O11W^ix^	0.82 (1)	1.93 (1)	2.747 (7)	175 (7)
O2W—H3W···O14W^iv^	0.82 (3)	2.06 (1)	2.865 (7)	168 (5)
O2W—H4W···O24^viii^	0.82 (2)	2.37 (7)	3.018 (6)	136 (8)
O3W—H5W···O8W^viii^	0.83 (4)	2.16 (6)	2.760 (6)	129 (6)
O4W—H7W···O14W^iv^	0.82 (3)	2.04 (3)	2.850 (6)	169 (5)
O4W—H8W···O7W^viii^	0.82 (3)	2.06 (2)	2.872 (6)	168 (9)
O5W—H9W···O23^ii^	0.82 (2)	2.41 (6)	2.937 (6)	123 (6)
O5W—H10W···O12^x^	0.83 (6)	2.45 (4)	3.179 (6)	149 (8)
O6W—H11W···O14^x^	0.82 (3)	2.15 (3)	2.874 (5)	147 (4)
O7W—H13W···O12^x^	0.82 (3)	2.11 (3)	2.878 (5)	157 (6)
O7W—H13W···O11^x^	0.82 (3)	2.55 (5)	3.036 (5)	119 (5)
O7W—H14W···O5^vi^	0.82 (6)	2.00 (5)	2.812 (6)	168 (9)
O8W—H15W···O14W^ix^	0.82 (2)	2.13 (3)	2.868 (6)	149 (5)
O8W—H16W···O4^vi^	0.83 (5)	2.05 (4)	2.808 (5)	154 (10)
O9W—H18W···O15^x^	0.82 (3)	2.21 (3)	3.021 (6)	172 (6)
O10W—H19W···O13W^ix^	0.82 (3)	2.06 (3)	2.877 (7)	176 (6)
O10W—H20W···O7^vi^	0.82 (2)	2.10 (2)	2.862 (5)	154 (5)
O11W—H21W···O1^i^	0.82 (2)	1.98 (3)	2.781 (5)	167 (5)
O11W—H22W···O23^x^	0.83 (4)	2.03 (3)	2.771 (5)	150 (6)
O13W—H25W···O20^ii^	0.82 (3)	2.55 (5)	2.918 (6)	109 (4)
O13W—H26W···O14^x^	0.81 (5)	2.23 (3)	2.935 (6)	144 (5)
O14W—H27W···O21^iii^	0.82 (1)	1.87 (2)	2.662 (5)	163 (6)
O14W—H28W···O8^xi^	0.82 (4)	1.96 (2)	2.761 (5)	167 (9)

Symmetry codes: (ix) −*x*+1, −*y*+1, *z*+1/2; (iv) −*x*+1, −*y*, *z*+1/2; (viii) *x*, *y*−1, *z*; (ii) −*x*+1/2, *y*, *z*−1/2; (x) −*x*+1, −*y*+1, *z*−1/2; (vi) *x*, *y*+1, *z*; (i) *x*+1/2, −*y*+1, *z*; (iii) −*x*+1, −*y*, *z*−1/2; (xi) −*x*+3/2, *y*, *z*−1/2.
